# Quantitative visualization of photosynthetic pigments in tea leaves based on Raman spectroscopy and calibration model transfer

**DOI:** 10.1186/s13007-020-00704-3

**Published:** 2021-01-06

**Authors:** Jianjun Zeng, Wen Ping, Alireza Sanaeifar, Xiao Xu, Wei Luo, Junjing Sha, Zhenxiong Huang, Yifeng Huang, Xuemei Liu, Baishao Zhan, Hailiang Zhang, Xiaoli Li

**Affiliations:** 1 College of Electrical and Automation Engineering, East China Jiaotong University, Nanchang, 330013 China; 2 College of Biosystems Engineering and Food Science, Zhejiang University, 866 Yuhangtang Road, Hangzhou, 310058 China; 3 College of Civil Engineering and Architecture, East China Jiaotong University, Nanchang, 330013 China

**Keywords:** Photosynthetic pigments, Concentration distribution imaging, Feature extraction, Model evaluation, Quantitative analysis, Raman spectroscopy

## Abstract

**Background:**

Photosynthetic pigments participating in the absorption, transformation and transfer of light energy play a very important role in plant growth. While, the spatial distribution of foliar pigments is an important indicator of environmental stress, such as pests, diseases and heavy metal stress.

**Results:**

In this paper, in situ quantitative visualization of chlorophyll and carotenoid was realized by combining the Raman spectroscopy with calibration model transfer, and a laboratory Raman spectral model was successfully extended to a portable field spectral measurement. Firstly, a nondestructive and fast model for determination of chlorophyll and carotenoid in tea leaf was established based on confocal micro-Raman spectrometer in the laboratory. Then the spectral model was extended to a real-time foliar map scanning spectra of a field portable Raman spectrometer through calibration model transfer, and the spectral variation between the confocal micro-Raman spectrometer in the laboratory and the portable Raman spectrometer were effectively corrected by the direct standardization (DS) algorithm. The portable map scanning Raman spectra of the tea leaves after the model transfer were got into the established quantitative determination model to predict the concentration of photosynthetic pigments at each pixel of the tea leaves. The predicted photosynthetic pigments concentration of each pixel was imaged to illustrate the distribution map of foliar pigments. Statistical analysis showed that the predicted pigment contents were highly correlated with the real contents.

**Conclusions:**

It can be concluded that the Raman spectroscopy was applicable for in situ, non-destructive and rapid quantitative detecting and imaging of photosynthetic pigment concentration in tea leaves, and the spectral detection model established based on the laboratory Raman spectrometer can be applied to a portable field spectrometer for quantitatively imaging of the foliar pigments.
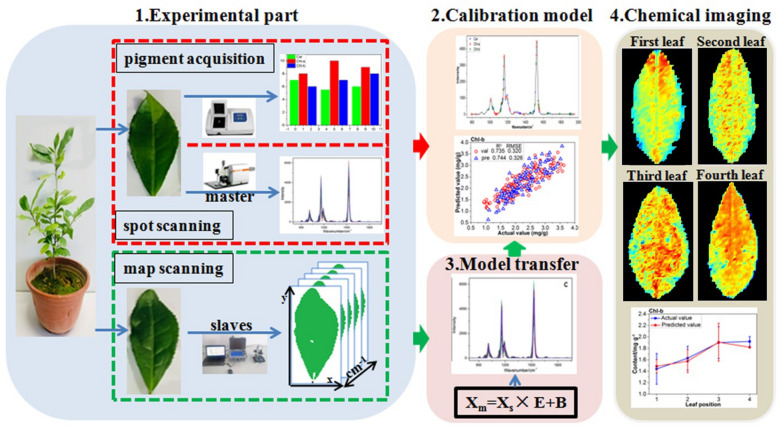

## Background

Tea is one of the world's three major beverages. The world's tea production exceeds 4 million tons per year and more than 2 billion people consume tea [1]. Tea, which contains high levels of antioxidants and can prevent many diseases including cardiovascular and cancer, has received more and more attention from people [2]. Photosynthesis is the determinant of productivity and the basis of plant growth and development, and an important source of carbon in plants. The photosynthesis of tea leaves is closely related to the quality and yield of tea. Photosynthetic pigments including chlorophyll a (Chl-a), chlorophyll b (Chl-b), and total carotenoids (Car) play a very important role in plant growth. Furthermore, the spatial distribution of foliar pigments is an important indicator of environmental stress, such as pests, diseases and heavy metal stress [3, 4]. In addition, the color and lustre of tea plays an important role in the consumption and production of tea, and the pigment concentration of tea is also an important factor affecting the color of tea. Therefore, developing a method for nondestructive detection and quantitative visualization of foliar photosynthetic pigment content is an important task for plant protection, cultivation and tea processing [5].

Most of the existing imaging studies on photosynthetic pigments concentration are based on visible-near infrared hyperspectral imaging technology. Previous studies have investigated crops such as cucumber leaf [6, 7], spinach [8], pepper leaf [9], tomato [10] and so on, and have achieved good results. Zhao et al. [11] used visible-near infrared hyperspectral technology to extract the corresponding feature parameters and built models based on 7 algorithms. The chlorophyll concentration was predicted by the models and the distribution map of chlorophyll concentration was drawn. Raman spectroscopy is a non-destructive analytical technique, which is based on the scattering interaction between light and chemical bond in materials. It can provide detailed information of chemical structure, phase and morphology, crystallinity and molecular interaction of samples. Compared to visible-near infrared hyper-spectrum, Raman spectroscopy has the advantages of high resolution, which can provide more spatial information for pest and heavy metal detection, and help to further study the mechanism of pests and heavy metal stress.

There are a lot of studies on the use of Raman spectroscopy to identify and visualize chemical compounds. Schulz et al. [12] used a NIR-FT-Raman spectrometer to detect carotenoid in various fruits and vegetables such as carrot, tomato, and nectarine, and found Raman spectral fingerprint peaks of carotenoid in plants. The characteristic peaks were used to image the carotenoid distribution inside the plants. Qin et al. [13] developed a bench-top point-scan Raman chemical imaging system to detect and visualize the internal distribution of lycopene in postharvest tomato, and established a Raman chemical image to visualize the spatial distribution of lycopene at different stages of maturity. Yang et al. [14] used a custom row-scan Raman hyperspectral imaging system to detect and display the main chemical components of maize seeds. The characteristic peaks associated with corn starch, mixture of oil and starch, zeaxanthin, lignin and oil were found. Each single band image corresponding to the characteristic band successfully represents the spatial distribution of chemical components in seeds.

In addition to the above qualitative analysis, Raman spectroscopy also has many applications in quantitative detection. Baranska et al. [15] detected lycopene and carotene in tomato and its products by FT Raman spectroscopy, and its modeling effect was better than that of near infrared spectrum but slightly lower than that of infrared spectrum. Bhosale et al. [16] and Dane et al. [17] detected the carotenoid concentration in kinds of fruits and vegetables (such as tomato, carrot, mango, etc.) using Raman spectroscopy. The result showed that there was a high correlation between the Raman spectrum signal intensity and the carotenoid concentration, and the correlation coefficient (R) was up to 0.9618. The above researches shows that Raman spectroscopy has great potential for quantitative detection of pigments, but the transferring of the quantitative model of Raman spectroscopy to portable devices, especially the field applicability of the laboratory model, has not been studied so far.

In practical applications of spectral determination model, it is often encountered that a multivariate calibration model developed based on one instrument (Master) cannot be used on another instrument (Slave) of the same type as the Master. Or there will be big biases in the prediction result. The poor adaptability of this spectral model greatly limits the application prospect of spectral detection technology, and the spectral models that took a lot of time and money to build in the laboratories are difficult to be used in field or production practice. A strategy of model transfer has been frequently adopted to solve this problem. The essence of model transfer is to overcome the inconsistency between the measured signals (or spectra) of samples on different instruments [18]. Chemometric techniques are used to correct the differences in instrumental response function and then making the existing model transferable [19]. Direct Standardization (DS) is the most widely used method among the calibration standardization methods. The DS method is usually preferred due to its ease of use in correcting the spectra [20]. In this method, the transformation matrix is achieved by modeling two batch spectra of standard samples dataset included on both instruments, and then correction between the new and original calibration datasets is performed, thereby predicting the transformed spectra of the new samples on the second instrument without loss of the accuracy of calibration models [21]. Linking the two instruments through model transfer not only can be applied in actual production, but also make the model more accurate. Ji et al. [22] used a direct standardization (DS) model transfer algorithm to remove the environmental factors from the field spectrum so as to effectively estimate the soil properties. Wang et al. [23] proposed a model transfer algorithm based on genetic algorithm, which makes the partial least squares model of aviation fuel density successfully transferred from one instrument to another, and the accuracy of model prediction result is close to the calibration model and higher than the DS model.

Confocal micro-Raman spectrometer has high resolution and precision, and can capture more useful spatial information, but this instrument is too expensive and heavy to be used in field or in vivo plant detection. Portable Raman spectrometer is compact and convenient, and can be used for in vivo or field detection, but it has the disadvantage of low resolution. In regards to field applications, Raman libraries are built and maintained on a more efficient laboratory instrument and then transferred to handheld/portable spectrometers. The need for a successful multivariate calibration model transfer that minimizes prediction error for a Raman spectral library database, such as those available and successfully applied extensively to near infrared (NIR) studies [24–27] has been a primary goal of the Raman spectroscopy measurements and has been the subject of some researches [28–31]. For instance, calibration transfer from an at-line to an in-line acquisition of Raman spectra was performed for PLS regression models to predict the concentration levels of two ingredients in a liquid detergent composition [19].

Therefore, further studies are needed to explore the possibility of creating calibration models on a laboratory-based Raman spectroscopy and transferring it to various field instruments. The calibration standardization procedures also provide the possibility for the building of standard free robust models. Nevertheless, little research on the measurement of photosynthetic pigments of tea leaf based on Raman spectroscopy was carried out, and there was no calibration transfer study in spatial distribution of foliar pigments, especially calibration transfer between tea leaf samples with different positions.

Good results have been obtained in the previous literatures, but the effect of model transfer method on the variation correction of Raman spectrometers has not been reported. Here we are committed to building a high-precision Raman spectral pigment measurement model based on high-performance instrument in the laboratory, and study the application of this spectral model to the portable field measurement equipment.

In order to solve the above problems, this paper aims to study the Raman spectral characteristics of tea leaf and establish an in situ quantitative analysis model between the concentration of photosynthetic pigment and its confocal micro-Raman spectra in tea leaf. On this basis, the portable Raman mapping data after model transfer were brought into the established model, the concentration of each pixel was predicted, and the chemical imaging was carried out to obtain the distribution map of the concentration of photosynthetic pigment in tea leaf at different position.

## Materials and Methods

### Materials and instruments

#### Material preparation

The tea variety is longjing 43 (*Camelliasinensis(L.) O.Kuntze*). In the summer of 2012, longjing 43 seedlings were obtained from the tea garden in Fuyang, Hangzhou, China. Tea seedlings were planted in pots in the agricultural internet of things exhibition center of college of biosystems engineering and food science, Zhejiang University, receiving natural light and artificial watering.

Material 1: in total of 315 leaves including the first four leaves in shoot (as shown in Additional file 1: Fig. S1) were plucked for Raman spectral collection, then all the leaves were stored in the refrigerator within 4 ℃ immediately. Following, approximately 0.1 g of weighed leaf, excluding central vein was taken for reference measurement of concentration of chlorophyll and total carotenoids according to the Reference [8] based on the ultraviolet spectrophotometer with the unit mg·g^−1^. In detail, 10 mL pigment extraction solution (95% alcohol solution) was added to the cut and ground sample, and stored in a dark room for about 24 h.

Material 2: in total of 16 leaves including 4 leaves in each position (as shown in Additional file 1: Fig. S1) were collected for different four tea plants. And the photosynthetic pigment concentration of the 16 leaves was measured in the same method as in the previous section.

#### Instrument

Master spot scanning: a laser confocal micro-Raman spectrometer (Renishaw, United Kingdom/Via-Reflex 532/XYZ) was used for collecting Raman spectra of the Material 1. Specific parameters were as follow: the excitation wavelength is 532 nm; the spectral collection range is 579–3062 Raman shift/cm^−1^ with a spectral resolution of 0.2 nm, the laser intensity is 50 mW, the exposure time is 1 s, and the objective lens multiple is 5. Each sample was collected at three points from top to base, and the average spectrum was used as the representative Raman spectrum of the sample. So, a total of 315 spectra were obtained.

Slave map scanning: a portable Raman spectrometer (Ocean optics QE Pro, United States) was used for spectral collection of Material 2. Specific parameters were as follow: the excitation wavelength is 532 nm, the spectral collection range is 77–2146 Raman shift/cm^−1^, the laser intensity is 100 mW, the exposure time is 3 s, and the average time is 2 s. The 16 leaves were map scanned by the Raman spectrometer with horizontal and vertical spatial resolution of 1 mm.

In this experiment, the software WIRE 3.3 (Renishaw, United Kingdom) was used to collect and extract Raman spectral data. The pictures in this paper were drawn by Origin 9.0 (Originlab, United States) and Photoshop CS6 (Adobe, United States), and all the preprocessing and modeling methods were performed in Matlab 2013b (MathWorks, United States).

### Spectral analysis methods

#### Spectral pretreatment

The obtained spectral information contains not only the chemical structure information of the sample, but also many background and noise signals from the interference source such as the instrument itself and the experimental operating environment. Therefore, in order to eliminate the influence of the extraneous and interfering signals on the sample signal, the original data can be preprocessed [32]. In this study, five data preprocessing methods were applied including multiplicative scatter correction (MSC), wavelet transform (WT), standard normal variate (SNV), rolling-circle filter (RCF) and adaptive iteratively reweighted penalized least squares (airPLS).

The full-band Raman spectrum contains a large amount of redundant information and noise [33]. These interference signal not only affect the prediction performance of the model, but also are not conducive to further detecting the Raman spectral response mechanism of chlorophyll. So, the competitive adaptive re-weighted algorithm (CARS) was used to extract the effective band for spectral measurement of the photosynthetic pigments. Based on the effective bands from CARS, the computational complexity of the spectral modeling can be reduced. Furthermore, the Raman spectral response mechanism of photosynthetic pigment may be discovered based on the assignment of these characteristic bands.

#### Sample division

The 315 samples from master spot scanning were divided into a training set and a test set based on 2:1 ratio. First, the samples were arranged in ascending order according to their concentrations of Chl-a, Chl-b and Car, and each three is one set in turn. Then, the second sample in each set is divi3ded into the test set, and the rest are set as the training set. So, 210 training set samples and 105 test set samples were obtained. The statistical information of the sample sets were shown in Table 1.

**Table 1:** The statistical information of the sample set

	Max (mg·g^−1^)	Min (mg·g^−1^)	Mean ± SD	Num
Car				
Training set	1.4917	0.1901	0.8461 ± 0.2282	210
Test set	1.4786	0.2557	0.8465 ± 0.2267	105
Chl-a				
Training set	10.4111	1.877	5.6513 ± 1.4116	210
Test set	10.4111	2.3599	5.6559 ± 1.4191	105
Chl-b				
Training set	5.7826	0.6288	2.2675 ± 0.6804	210
Test set	5.7826	0.9380	2.2785 ± 0.7128	105

*Max* maximum value, *Min* minimum value, *SD* standard deviation, *Num* number of samples

#### Modeling and evaluation methods

Partial least squares regression (PLSR) was adopted to establish a quantitative relationship between the concentration of photosynthetic pigments and Raman spectra of leaf. The PLSR has the advantages of simplicity, accuracy, convenience and wide applicability. It is the most commonly used and most effective multivariate statistical method in chemometric modeling analysis [34]. The performance of the PLSR model was evaluated by the following indicators including coefficient of determination (R^2^) and root mean square error (RMSE) [35]. In detail, the R^2^_C_, R^2^_CV_, R^2^_P_ and RMSE_C_, RMSE_CV_, RMSE_P_ represent the determination coefficient and root mean square error of calibration, cross validation and prediction, respectively.

Considering the subsequent model transfer, the spectral bands of the two instruments need to be unified. The common band range of the two spectrometers is 579–2146 cm^−1^, but there are obvious high-frequency noise signals at the both ends. So the spectral range of 792–1961 cm^−1^ was selected for modeling.

### Model transfer

In order to test the prediction result of the model, the sample after the scanning should measure 3 photosynthetic pigments concentration, and compare the actual value with the predicted value. Scanning takes a long time, and if it is detected in vitro, it will inevitably affect the accuracy of photosynthetic pigment concentration. Although the master also has a scanning function, its structure is complicated and impossible to perform living body detection of tea leaves. The slave is small, portable, simple in structure, and can perform scanning without removing the tea leaves. Therefore, the slave is used to scan four leaves of different leaf positions.

The direct standardization (DS) method was adopted to improve the adaptability of the spectral model, which is a multivariate full-spectrum model transfer algorithm [36]. The advantage is that the principle is simple, the difference between the standard spectral data and the spectral data to be corrected can be compared, and each wavenumber is sequentially corrected by the full band to realize the transfer of the model [23].

The common spectral range of 579–2146 cm^−1^ of the two Raman spectrometers was selected, and to remove the high-frequency noise signals at both ends, the range of 792–1961 cm^−1^ was selected for further model transfer. Since the spectral resolutions of the two spectrometers are different, interpolation processing was performed to form a uniform number of spectral variables. It is important for model transfer to choose representative samples to define the differences between the master and slave instruments. For the slave instrument, the average spectrum of all pixels for each leaf (minus the background) map scanning was taken as the representative spectrum of the leaf, so a total of 16 spectral profiles were obtained. And 4 representative spectra were selected by the Kennard-Stone (KS) algorithm from the 16 spectra. While, for the master instrument, 4 spectra were also selected by KS algorithm from the 315 spectra of master spot scanning. Model transfer was performed by selecting 4 spectra (including all of the leaf positions) from the master and slave instrument.

The flow of the DS algorithm is as follow [37, 38]. The spectral matrices of master and slave are X_m_, X_s_, respectively. Both X_m_ and X_s_ have size m × p, where m represents the number of representative transfer spectra (4 in this case) and p represents the number of wavenumbers.

1 $${\mathrm{X}}_{\mathrm{m }}= {\mathrm{X}}_{\mathrm{s}}\times \mathrm{E}+\mathrm{B}$$

where E is the transfer matrix with size p × p of unknown parameter, which accounts for the variation in both X_m_ and X_s_, and B is the background correction matrix [22].

The spectrum S_s_ of sample to be tested measured on the slave can be used for analysis after conversion:

2 $${\mathrm{S}}_{\mathrm{s},\mathrm{ std}}={\mathrm{S}}_{\mathrm{s}}\times \mathrm{E}+\mathrm{B}$$

The spectral data S_s,std_ represents the corrected slave spectra through transferred by the DS algorithm, which will be transmitted to the photosynthetic pigments determination model developed by the master. And the prediction accuracy of the model for the slave sample is the performance of model transfer.

## Results and discussion

### Establishment of quantitative determination model

#### Raman spectral quantitative determination of photosynthetic pigments in tea leaf

As shown in Table 2, different pretreatment methods produced different results referring to the values of R^2^ and RMSE, indicating that pretreatment has a great influence on the performance of the model. In regard of the Car, model 5 based on the WT preprocessing method is obviously better than model 1 based the original data. In detail, the R^2^_p_ of the model 1 increased from 0.614 to 0.713 of the model 5, and RMSE_p_ of the model 1 decreased from 0.140 to 0.108 of the model 5. For Chl-a and Chl-b, model 10 and model 16 respectively obtained the best results based on the optimal pretreatment method of RCF. Comparing with the model 7 based on the original data, the R^2^_p_ of model 10 increased from 0.597 to 0.800, and RMSE_p_ of model 10 decreased from 0.900 to 0.599. While, the R^2^_p_ and RMSE_p_ of model 16 were respectively 0.734 and 0.330, which were obviously better than the relevant parameters (0.718 and 0.342) of model 13. Furthermore, the difference among calibration, validation and prediction of the model 5, 10 and 16 was also relatively small, which indicates that the stability of these models is improved through pretreatment.

**Table 2:** PLSR modeling results of different pretreatment methods

	**Model**	**Pretreatment**	**Calibration**		**Validation**		**Prediction**	
			**RMSE**_**C**_	**R**^**2**^_**C**_	**RMSE**_**CV**_	**R**^**2**^_**CV**_	**RMSE**_**P**_	**R**^**2**^_**P**_
Car	1	Origin	0.082	0.843	0.107	0.732	0.140	0.614
	2	MSC	0.080	0.847	0.106	0.738	0.134	0.650
	3	SNV	0.080	0.848	0.105	0.744	0.135	0.642
	4	RCF	0.122	0.653	0.142	0.532	0.131	0.662
	5	WT	0.086	0.826	0.106	0.737	0.108	0.713
	6	airPLS	0.123	0.646	0.140	0.549	0.199	0.227
Chl-a	7	Origin	0.463	0.870	0.577	0.798	0.900	0.597
	8	MSC	0.557	0.812	0.692	0.712	0.867	0.626
	9	SNV	0.834	0.650	0.898	0.600	0.886	0.609
	10	RCF	0.535	0.827	0.653	0.745	0.599	0.800
	11	WT	0.095	0.806	0.116	0.715	0.108	0.721
	12	airPLS	0.622	0.773	0.820	0.615	1.078	0.421
Chl-b	13	Origin	0.240	0.854	0.300	0.774	0.342	0.718
	14	MSC	0.284	0.784	0.339	0.695	0.366	0.677
	15	SNV	0.282	0.789	0.343	0.690	0.353	0.701
	16	RCF	0.295	0.810	0.347	0.717	0.330	0.734
	17	WT	0.299	0.772	0.349	0.690	0.354	0.698
	18	airPLS	0.247	0.844	0.340	0.707	0.385	0.644

#### Selecting of characteristic bands for quantitative determination

In the present study, WT and RCF pretreatment methods have improved spectral data with higher R^2^_P_ value and lower RMSEP when compared to other methods (Table 2). The analysis of Raman spectra is usually involved with background problems caused by fluorescence effects.

Raman spectroscopy is a weak scattering signal, which intensity is about 1/10 million of that of Rayleigh scattering, that often accompanies it. And it is particularly easily interfered by the background fluorescence of plant tissue, which makes it difficult to directly use the spectrum for reliable quantitative and positional analysis [39]. The background should be deleted because there is no chemical information in it. RCF is an easy-to-use and intuitive filter to eliminate background effects [40]. According to the results reported by [41–43], the background with minimum changes in the parameters of the Raman spectra is effectively subtracted by the RCF method. Due to its advantages, this method was widely used and previous research results in the field of Raman spectroscopy proved that RCF is superior to other methods, which is consistent with the results obtained in this work.

WT is also a very powerful tool in compressing analytical signals [44, 45]. It transforms the raw data into the wavelet domain, so the information included in raw data can be compressed and explained by a small number of wavelet coefficients. WT was successfully applied and multivariate analytical problems were significantly simplified by this method [46, 47].

Raman spectroscopy provides a wide range of spectral information. In this research, there are 1005 and 1044 spectral variables from the master and the slave instruments respectively. And, there are still remaining 448 spectral variables after intercepting the common wavenumbers and removing the two ends of the spectrum seriously disturbed by noise. The Raman spectra contain not only biological, physiological and structural information related to detection objects, but also redundant information [33]. In order to explore the mechanism of the detection of photosynthetic pigment in tea leaves by Raman spectroscopy, a large number of redundant and interference information were excluded. Furthermore, selecting a small number of effective band can shorten the modeling time and improve the accuracy of the model. The CARS was adopted to extract the effective band for spectral measurement of the photosynthetic pigments based on the spectral data pretreated by the WT and RCF pretreatment, and the models based on the characteristic bands were established, and the modeling results were shown in Table 3. It can be found that the RCF was better than the WT pretreatment for all the three pigments, and the models based on these characteristic bands were better compared with the full-band models (as shown in Table 2).

**Table 3:** PLSR modeling results based on characteristic band

	**Model**	**Number of characteristic bands**	**Pretreatment**	**Calibration**		**Validation**		**Prediction**	
				**RMSE**_**C**_	**R**^**2**^_**C**_	**RMSE**_**CV**_	**R**^**2**^_**CV**_	**RMSE**_**P**_	**R**^**2**^_**P**_
Car	19	40	RCF	0.109	0.739	0.124	0.669	0.101	0.769
	20	40	WT	0.125	0.702	0.133	0.661	0.123	0.706
Chl-a	21	37	RCF	0.586	0.793	0.623	0.767	0.517	0.852
	22	37	WT	0.603	0.782	0.777	0.753	0.623	0.835
Chl-b	23	32	RCF	0.298	0.769	0.320	0.735	0.326	0.744
	24	32	WT	0.343	0.746	0.443	0.708	0.389	0.726

**Fig. 1:**
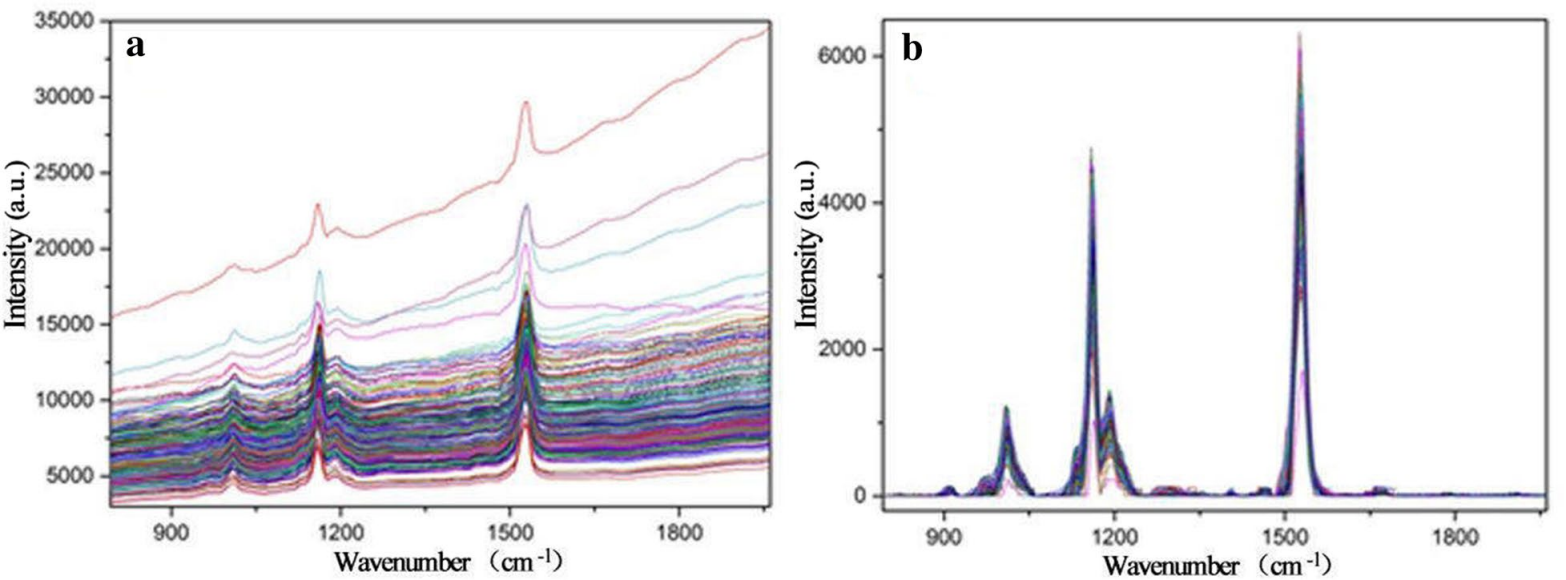
Raman spectral profiles of tea leaf samples from master instrument. **a** original spectra; **b** spectra processed by the RCF

The spectral profiles before and after the RCF pretreatment were shown in Fig. 1, it can be found that the RCF method eliminated the fluorescence background and increased the signal-to-noise ratio of spectra, this may be the reason why the RCF pretreatment can improve the performance of the spectral determination models. Oh et al. [48] used RCF pretreatment in real-time estimation of glucose concentration in algae by Raman spectroscopy, and the result was also improved.

**Fig. 2:**
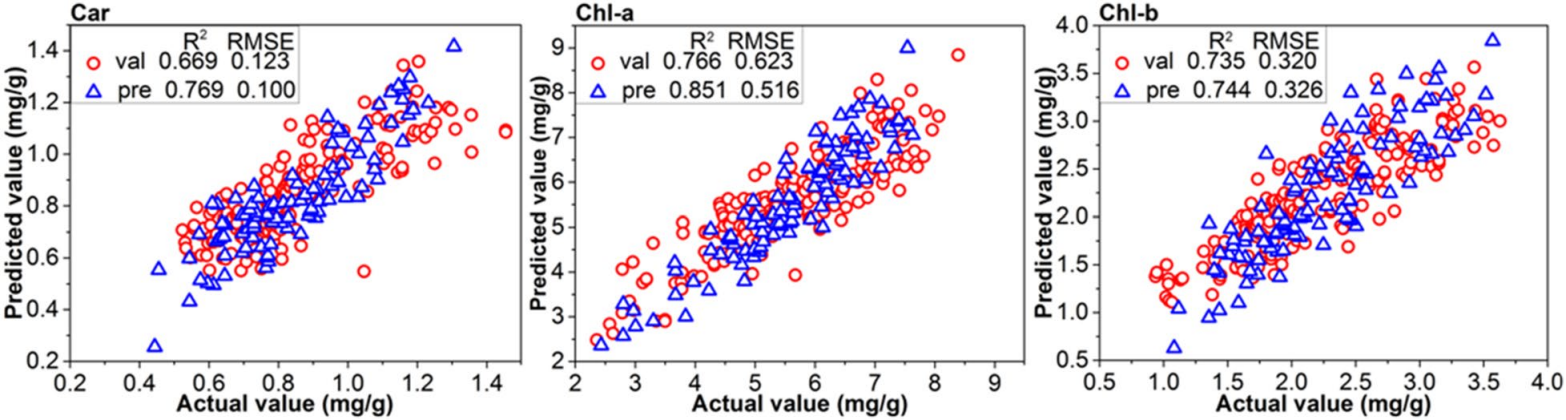
PLSR model results achieved from the master instrument based on the CARS characteristic bands selection for **a** total carotenoids, **b** chlorophyll a, **c** chlorophyll b (*Val* validation set and *Pre* prediction set)

Scatter diagram of prediction values and real values of the models (model 19, 21 and 23) for training and test samples were shown in Fig. 2. It can be found that the models based on the characteristic wavenumbers had achieved better result than the model based on the full-band model (as shown in Table 2). In addition, low dimensional input variables of characteristic wavenumbers greatly reduce the complexity of the model and improve the calculation speed of the model. Zhao et al. [11] used hyperspectral imaging technique to build the models to estimate the chlorophyll content in tea and obtained RMSE_C_, R^2^_C_, RMSE_P_ and R^2^_P_ of Chl-b model with the values of 9.918, 0.711, 8.601, and 0.693, respectively, which is obviously worse than the result of our research. Therefore, the obtained results showed that it is feasible to predict the concentration of photosynthetic pigments based on Raman spectroscopy.

**Fig. 3:**
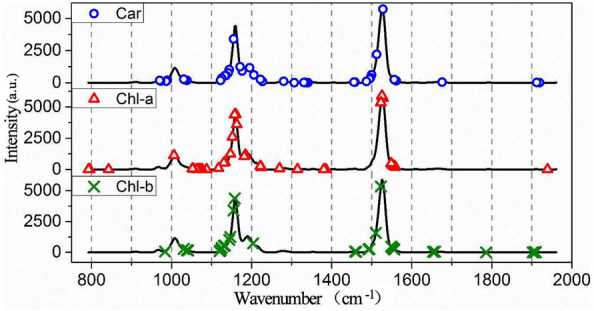
Distributions of the characteristic wavenumbers selected based on CARS algorithm

As the Raman spectroscopy can reflect the fingerprints information of the composition and structure of substances, an assignment of these characteristic wavenumbers was implemented to further explore the substance basis of quantitative determination of pigment by Raman spectroscopy. The Raman spectral characteristic bands for pigments detection were selected by the CARS algorithm and the selected wavenumbers were shown in Fig. 3 and Additional file 2: Table S1. There were three distinct peaks in the figure, including the rocking vibration in the CH_3_ plane at 1008 cm^−1^, the C–C stretching vibration at 1159 cm^−1^, and C = C stretching vibration at 1528 cm^−1^ which are the characteristic peaks of photosynthetic pigment [49, 50]. The assignment of these characteristic wavenumbers was shown in Table 4, and the wavenumbers were connected to the composition and structure of substances based on the references. It can be seen that most of the wavenumbers were related to photosynthetic pigment, which explains the reason why models based on characteristic wavenumbers obtained good results.

**Table 4:** Chemical assignment of Raman characteristic wavenumbers

Wavenumbers/cm^−1^	Assignment	References
788/800/1046/1073/1114/1222/1553	Chlorophyll a	[51]
971	β-carotene [τ(C_11_-C_12_)]	[52]
988	Chlorophyll	[53]
1026	β-carotene [ρ(9Me), ν(C_8_ − C_9_)]	[52]
1068/1155/1165/1265/1392/1530/1555	Chlorophyll a	[54]
1128/1160/1210/1380/1465/1523/1567/1644	Chlorophyll b	[54]
1133	β-carotene	[55]
1137	β-carotene [ν(C_10_ − C_11_)]	[52]
1144	Chlorophyll [ν(CN), δ(CNC)]	[53]
1145	Chlorophyll a [ν(C_a_N), δ(C_a_NC_a_)]	[52]
1149	β-carotene [14 − 15, 15H, 15 = 15 ‘]	[55]
1157	β-carotene [ν(C_14_ − C_15_), δ(C_15_ − H)]	[52]
1172	β-carotene [5 − 4, 18r, 6 − 7]	[55]
1186	Chlorophyll a [ν(C_m_C_10_), δ (C_b_H)]	[52]
1187	β-carotene [10H, 11H, 8 − 9]	[55]
1191	β-carotene [δ(C_10_ − H), δ(C_11_ − H)]	[52]
1216	β-carotene [ν(C_12_ − C_13_), δ(C_14_ − H)]	[52]
1225	Chlorophyll [δ(CH), δ(CH_2_)]	[53]
1226	β-carotene [12 − 13, 14H, 15 = 15 ‘]	[54]
1281	β-carotene [15H, 14H, CCC15b]	[55]
1310	β-carotene [12H, 11 = 12, 15 = 15 ‘]	[55]
1322	β-carotene [δ(C_12_ − H), ν(C_11_-C_12_)]	[52]
1347	β-carotene [12H, 11 = 12, 15 = 15 ‘]	[55]
1450	β-carotene [δas(9Me), δas(13Me)]	[52]
1485	β-carotene [13 = 14, 11 = 12, 12H]	[55]
1518	Lutein [C=C stretching vibration]	[56]
1524	Chlorophyll a [ν(C_b_C_b_), ν(C_a_C_b_)]	[52]
1528	Carotenoids [ν1(C=C)]	[52]
1542	Chlorophyll a [ν(C_b_C_b_)]	[52]
1552	Chlorophyll a [ν(C_a_C_b_), ν(C_b_C_b_)]	[52]
1562	β-carotene [11 = 12, 15H, 12H]	[55]

In addition, several characteristic wavenumbers extracted in this study were also related to protein (1651 cm^−1^) and nucleic acid (1665 cm^−1^) [56], etc., this may be due to that the concentration of photosynthetic pigments is the percentage of the amount of pigment to the mass of dry matter in tea leaves, in other words, the quantity of other dry matter in tea will also affect the percentage of pigment, so the characteristic peaks of other dry matter in the tea will also be selected. Furthermore,

the wavelength selection algorithm based on data mining may also select some bands without specific component assignment as a benchmark for data processing.

### Calibration model transfer

#### Direct standardization of spectral data from the master and slave instruments

**Fig. 4:**
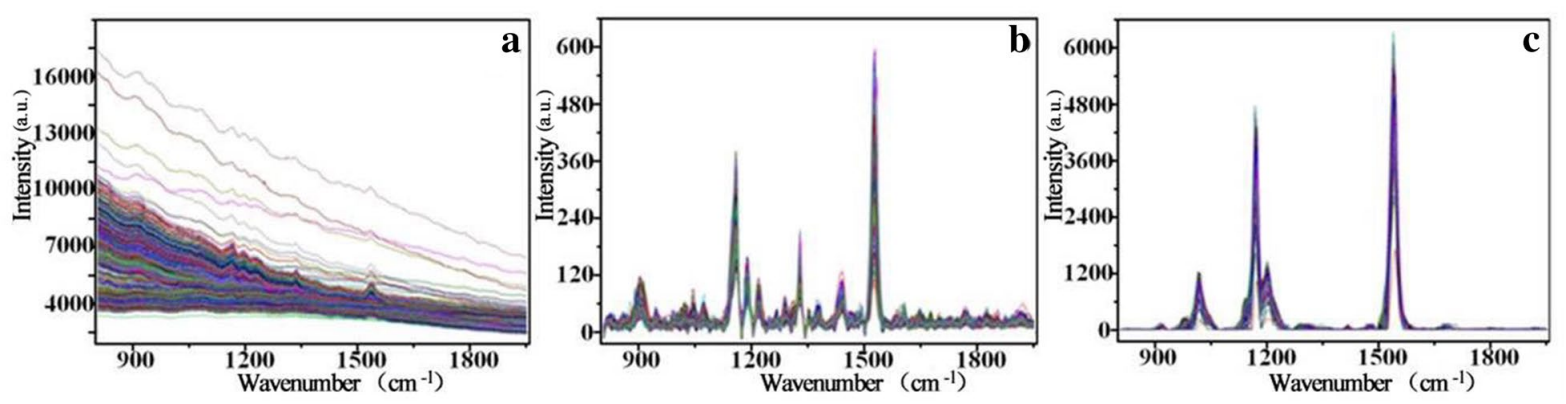
Raman spectral profiles of tea leaf samples from slave instrument. **a** original spectra; **b** spectra processed by the RCF; **c** spectra after DS

The direct standardization (DS) method was adopted to standardize the Raman spectral responses from the slave instrument. It can be seen from the Fig. 1(a) and Fig. 4(a) that the spectral data measured by the two instruments all had distinct fluorescent background, and the trend of the fluorescent background was different due to the different instruments. The spectra after removing the fluorescent background by using RCF was respectively shown in Fig. 1(b) and Fig. 4(b), it can be found that the RCF pretreatment greatly improves the signal to noise ratio of the spectra, which is conducive to the subsequent analysis. Slave spectral after DS was shown in Fig. 4(c). Comparing with Fig. 1(b), it can be found that the spectra of slave instrument (as shown in Fig. 4(c)) after DS was similar to that of the master instrument, indicating that the spectral variation between the master and slave Raman spectrometer can be effectively eliminated.

#### Imaging photosynthetic pigment in tea leaf based on model transfer

**Fig. 5:**
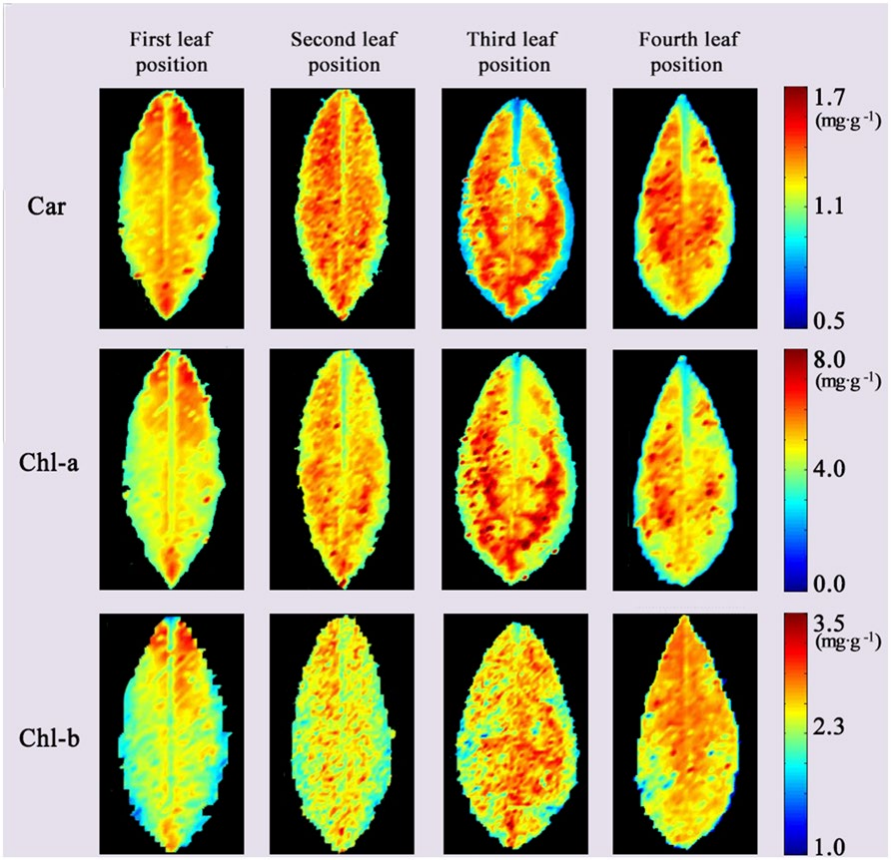
Distribution maps of photosynthetic pigment concentration in different leaf positions based on the slave instrument after calibration model transfer

Through the above analysis, the quantitative relationship between photosynthetic pigment concentration in fresh leaves and there’re master Raman spectroscopy had been verified, and the quantitative determination models of chlorophyll and carotenoid concentration based on characteristic wavenumbers had been established. In order to realize the in situ and non-destructive imaging of chlorophyll and carotenoid concentration in fresh leaves of tea, the slave Raman spectra of material 2 after DS were transported into the established model 19, 21, 23, respectively in pixel-wise order, so the photosynthetic pigment concentration of each pixel in tea leaf was predicted. The predicted photosynthetic pigment concentration was imaged, and the image was subjected to filter filtering to obtain distribution maps of photosynthetic pigments as shown in Fig. 5.

By imaging the photosynthetic pigments concentration, it can be found that the pigments concentration in the central vein and margin of the leaf is significantly lower than that in other region, which is related to the maximum efficiency of photosynthesis. These findings are consistent with the results found by [57].

#### Evaluation of performance of the calibration model transfer

After the map scanning spectra of the slave spectrometer were corrected, the spectrum at each pixel was brought into the quantitative determination model to predict the photosynthetic pigment concentration at that pixel. Then the photosynthetic pigment concentration of the foliar pixels was averaged to represent the pigment concentration of the leaf. Furthermore the predicted average value of pigment concentration was compared with the actual value to evaluate the performance of this calibration model transfer, in detail, the R^2^ and RMSE were shown in Additional file 3: Fig. S2. It can be found that the predicted value of the model for the foliar map scanning spectra is highly correlated with the actual value, which indicates that the pigment determination model based on the master instrument can predict the spectrum of the slave instrument after calibration model transfer. The imaging of foliar pigments results and the correlation analysis proved that the model transfer of the two spectrometers had achieved good results, and this method is feasible. Furthermore, the spectral calibration model constructed in the laboratory (master Raman spectrometer) can be used to measure the distribution of foliar pigment with portable instruments (slave) in the field through model transfer.

It is worth noting that the values obtained for the master instrument correspond to point scanning, and only three spectra are taken from each leaf. Although the spatial resolution of the slave instrument is less than that of the master instrument, the slave spectra are obtained by surface scanning, with hundreds or thousands of spectral lines per leaf, so it more closely corresponds to the chlorophyll in the leaf. In addition, slave spectra become very similar to master spectra through data processing methods such as model transfer.

As for the DS spectral correction method, a subset of samples that represented the entire experimental dataset well was required to measure the difference in the response of spectra measured under different instruments. Also, too few or too many samples in the transfer set can lead to under or over fitting, this implies that the predictive power of the model has not improved in terms of precision. So, further investigation with more samples in the calibration set or exploring another way to optimize the parameters is necessary for reliable use of the proposed method. The better performance of the slave instrument compared to the master instrument in some of the models transferred is consistent with the previous literature [20, 25, 58].

**Fig. 6:**
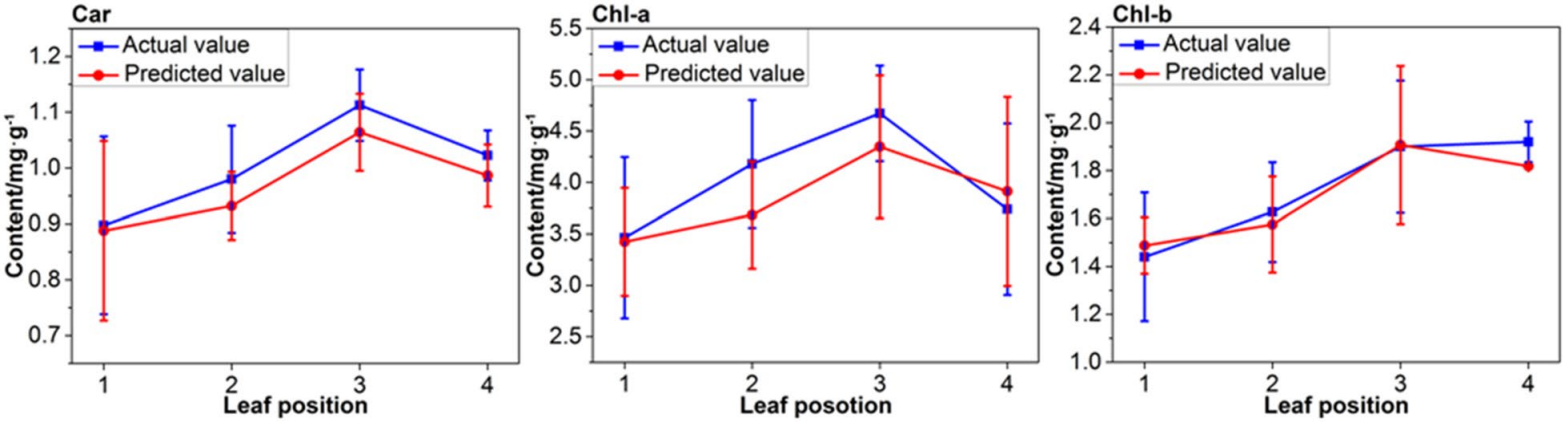
Line chart of actual and predicted values of photosynthetic pigment concentration achieved from the slave instrument after calibration model transfer

The mean and variance of the actual and predicted value of photosynthetic pigment in four leaves of the same leaf location were calculated, as shown in Fig. 6. As can be seen from the Fig. 6, the trend of the actual value of photosynthetic pigment increase firstly and then decrease, this is consistent with the finding of Vicente et al. [59]. The concentration of pigments at the first leaf position is low due to poor photosynthesis. The level of photosynthetic pigments increases with an increase in leaf position (age) and growing leaves. The photosynthesis rate reaches the highest value when the leaves are mature (third leaf position), and then decreases substantially during senescence due to weakening in the ability of photosynthetic enzyme expression [9]. The result show that the model established in this paper also has a prospect in the study of the leaf position and leaf age.

## Conclusions

In the study, the potential of Raman spectroscopy for in situ, non-destructive and rapid quantitative detection and imaging of photosynthetic pigment concentration in tea leaves was proved. Based on the Raman spectral pretreatment method combined with the CARS characteristic bands selection, the quantitative determination models of chlorophyll and carotenoid concentration were established by regression analysis. By comparison, it can be found that the best pretreatment RCF was most suitable to eliminate the fluorescence interference and other noise in Raman spectrum. And the Raman spectral characteristic bands for pigments detection selected by CARS have been proved to be the Raman-active molecular vibration of pigment components.

In addition, model transfer method was applied to the master and slave spectrometers in order to obtain a model that can be available both in vivo or in the field and with high prediction accuracy. This is the first attempt to establish a connection between the two types of instruments, and achieved good results. The foliar map scanning spectra after DS was brought to the pigments determination model established based on master instrument, and the concentration of photosynthetic pigment of each pixel in tea leaves could be predicted. Calculating the R^2^ between the predicted value and the actual value, the range was in the range of 0.752–0.866. The predicted chlorophyll and carotenoid concentration of each pixel were imaged to obtain the distribution map of photosynthetic pigment in tea leaves, illustrating that the concentration of photosynthetic pigment in the central vein and leaf margin was lower than other parts was obtained, which is consistent with other studies. In the future, this algorithm can be applied to calibration transfer in other plants with different types of samples, different geographical regions, and different varieties to establish more robust models with more physiological and biochemical indexes (e.g., moisture content, mineral elements, dry matter, etc.), which provide a technological basis for the effective detection of the growth and nutrient distribution in plants. Also, further studies will be related to the effect of the data size and optimization sets on the model transferability and comparison of different model transfer algorithms to discuss the results of calibrations.

It is worth noting that we have successfully improved the applicability of the Raman spectral model for determination of photosynthetic pigments in tea leaf. Through the calibration model transfer, the tea pigment spectral detection model based on the laboratory spectrometer was successfully applied to the portable quantitative detection of leaf pigment in the field. The model transfer method can effectively eliminate the spectral variation between the master and slave Raman spectrometers and improve the applicability of the spectral model, which will greatly promote the application process of the nondestructive and fast spectra measurement technique.

## Supplementary information

**Additional file 1: Fig. S1.** Leaf position of tea samples.

**Additional file 2: Table S1.** Characteristic wavenumbers selected by CARS algorithm for photosynthetic pigments in tea leaf.

**Additional file 3: Fig. S2.** Scatter diagram of actual and predicted values of photosynthetic pigment concentration.

## Data Availability

All the seed material and raw imaging data can be obtained from the authors upon request.

## Abbreviations

DS: Direct standardization
Chl-a: Chlorophyll a
Chl-b: Chlorophyll b
Car: Total carotenoids
CSM: Multiplicative scatter correction
WT: Wavelet transform
SNV: Standard normal variate
RCF: Rolling-circle filter
airPLS: Adaptive iteratively reweighted penalized least squares
CARS: Competitive adaptive re-weighted algorithm
PLSR: Partial least squares regression
R^2^: Coefficient of determination
RMSE: Root mean square error
KS: Kennard-Stone
