# Supplementary figures and images for: Quantitative visualization of photosynthetic pigments in tea leaves based on Raman spectroscopy and calibration model transfer
Source: Plant Methods. 2021 Jan 6;17:4. doi: 10.1186/s13007-020-00704-3 (PMC7788994; doi:10.1186/s13007-020-00704-3)

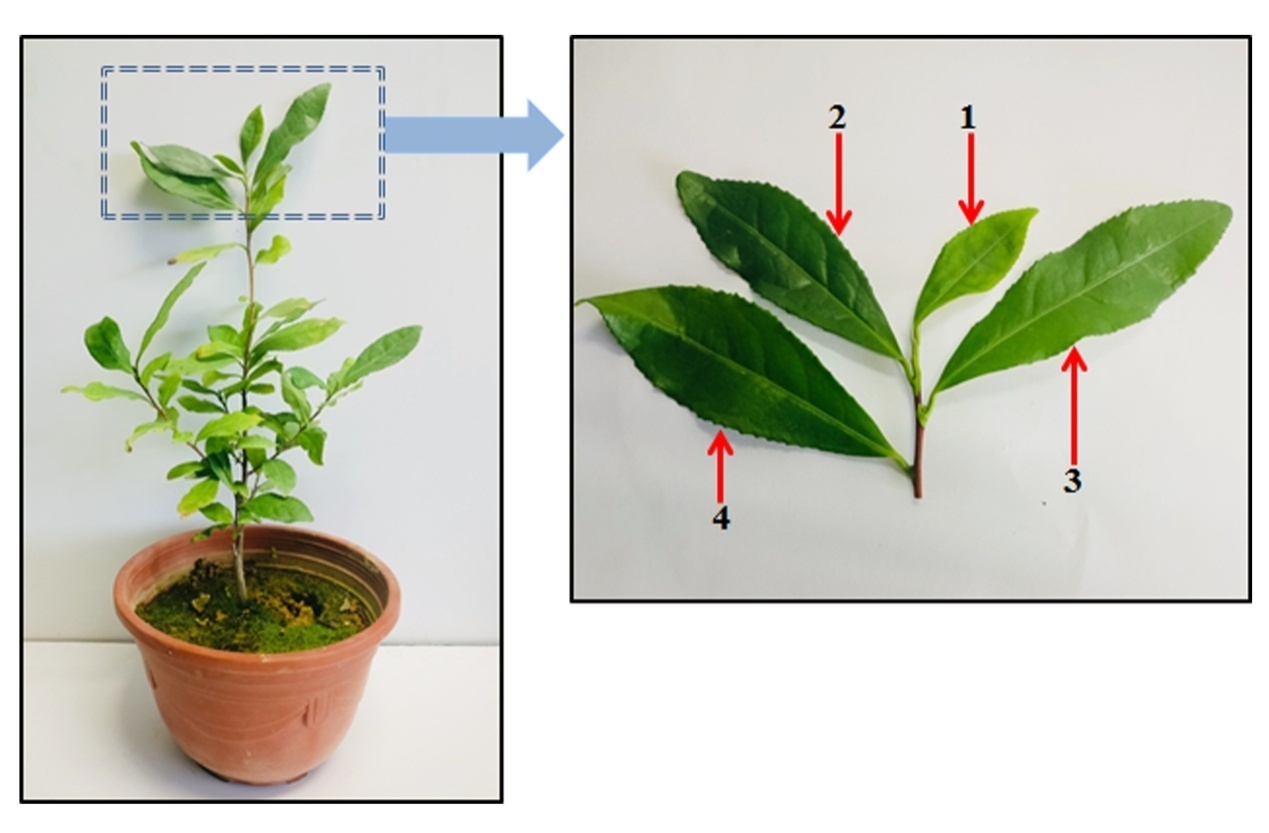


Fig. s1. Leaf position of tea samples.

Supplement: Supplementary file 1 — Additional file 1: Fig. S1. Leaf position of tea samples. [file 13007_2020_704_MOESM1_ESM.docx]
