# Supplementary material for: Quantitative visualization of photosynthetic pigments in tea leaves based on Raman spectroscopy and calibration model transfer
Source: Plant Methods. 2021 Jan 6;17:4. doi: 10.1186/s13007-020-00704-3 (PMC7788994; doi:10.1186/s13007-020-00704-3)
Supplement: Supplementary file 2 — Additional file 2: Table S1. Characteristic wavenumbers selected by CARS algorithm for photosynthetic pigments in tea leaf. [file 13007_2020_704_MOESM2_ESM.docx]

Table s1. Characteristic wavenumbers selected by CARS algorithm for photosynthetic pigments in tea leaf

|  | Characteristic wavelengths (cm^-1^) |
| --- | --- |
| Car | 970, 972, 986, 989, 1030, 1033, 1038, 1122, 1125, 1133, 1136, 1141, 1144, 1154, 1170, 1176, 1195, 1205, 1221, 1227, 1280, 1306, 1330, 1333, 1335, 1338, 1340, 1455, 1458, 1486, 1494, 1497, 1499, 1512, 1528, 1556, 1561, 1675, 1912, 1919 |
| Chl-a | 792, 795, 842, 1005, 1052, 1054, 1062, 1065, 1068, 1071, 1076, 1087, 1117, 1130, 1133, 1146, 1152, 1157, 1160, 1162, 1181, 1184, 1221, 1224, 1269, 1314, 1380, 1385, 1522, 1525, 1528, 1548, 1551, 1553, 1556, 1558, 1939 |
| Chl-b | 983, 1030, 1038, 1041, 1119, 1122, 1125, 1130, 1133, 1144, 1146, 1154, 1157, 1203, 1458, 1460, 1491, 1494, 1510, 1522, 1548, 1551, 1553, 1556, 1652, 1655, 1658, 1786, 1902, 1904, 1907, 1909, |
