# Supplementary material for: Quantitative visualization of photosynthetic pigments in tea leaves based on Raman spectroscopy and calibration model transfer
Source: Plant Methods. 2021 Jan 6;17:4. doi: 10.1186/s13007-020-00704-3 (PMC7788994; doi:10.1186/s13007-020-00704-3)
Supplement: Supplementary file 3 — Additional file 3: Fig. S2. Scatter diagram of actual and predicted values of photosynthetic pigment concentration. [file 13007_2020_704_MOESM3_ESM.docx]

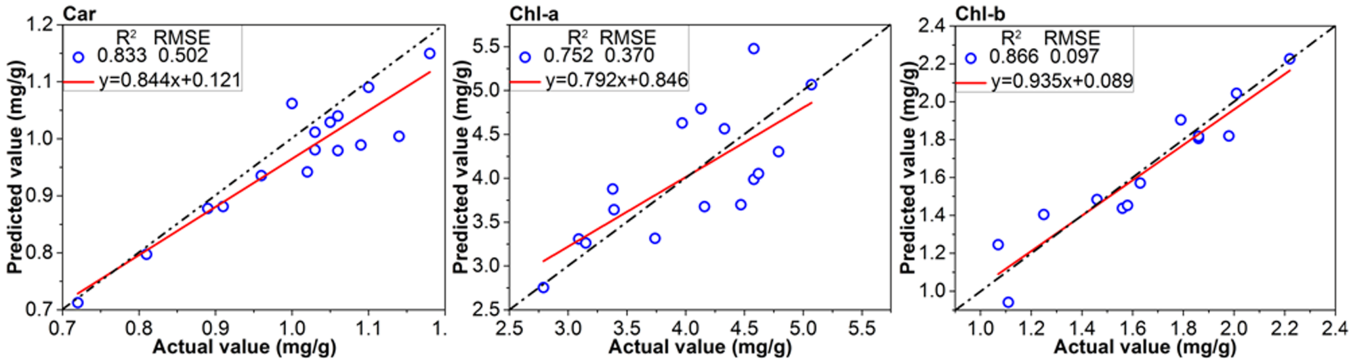


Fig. s2. [Scatter](javascript:;) [diagram](javascript:;) of actual and predicted values of photosynthetic pigment concentration.
